# Bringing inorganic chemistry to life with inspiration from R. J. P. Williams

**DOI:** 10.1007/s00775-016-1333-3

**Published:** 2016-02-03

**Authors:** H. Allen O. Hill, Peter J. Sadler

**Affiliations:** Inorganic Chemistry Laboratory, South Parks Road, Oxford, OX1 3QR UK; Department of Chemistry, University of Warwick, Gibbet Hill Road, Coventry, CV4 7AL UK

**Keywords:** Irving-Williams series, Metalloproteins, Vitamin B_12_, Entatic state, The metallome, Platinum drugs, Essential elements

## Abstract

Our appreciation of the scholarly ideas and thinking of Bob Williams is illustrated here by a few of the areas in which he inspired us. His journey to bring inorganic chemistry to life began with an early interest in analytical chemistry, rationalising the relative stabilities of metal coordination complexes (*The Irving*-*Williams Series*), and elucidating the organometallic redox chemistry of vitamin B_12_. He (and Vallee) recognised that metal ions are in energised (*entatic*) states in proteins and enzymes, which themselves are dynamic structures of rods and springs. He played a key role in helping Rosenberg to pave the road toward the clinic for the anticancer drug cisplatin. He believed that evolution is not just dependent on DNA, but also on the metallome. Organisms and the environment are one system: does DNA code directly for all the essential elements of life?

Witness a young speaker giving a seminar in 1948. An invited guest is Linus Pauling, probably the most famous chemist in the world. The young man was talking about his research, which was concerned with the stability constants of divalent metal ions with various ligands. They seemed to form a series: Mn^2+^ < Fe^2+^ < Co^2+^ < Ni^2+^ < Cu^2+^ > Zn^2+^. As was then customary in the USA, but not in the UK, Pauling asked a question of the speaker during the lecture, who said that he would answer the enquiry *after* the lecture. When Pauling asked the Chairman, “Who was that post-doctoral student who was speaking?” he was told that he wasn’t a post-doc, not even a graduate student, but still an *under*graduate! The order of the ions became known [1] as the Irving-Williams series.

R. J. P. Williams or, as he was always known, Bob Williams, was a man who *enjoyed* thinking (Fig. 1). Not necessarily with some action in mind, but the very act of cogitation gave him pleasure. In a very perceptive review [2] in 1953 entitled “Metal ions in biological systems,” he considered the whole subject of metal complexes, including their role in catalysis and especially enzyme activity. It is amazing how perceptive the conclusions are to this article:

**Fig. 1:**
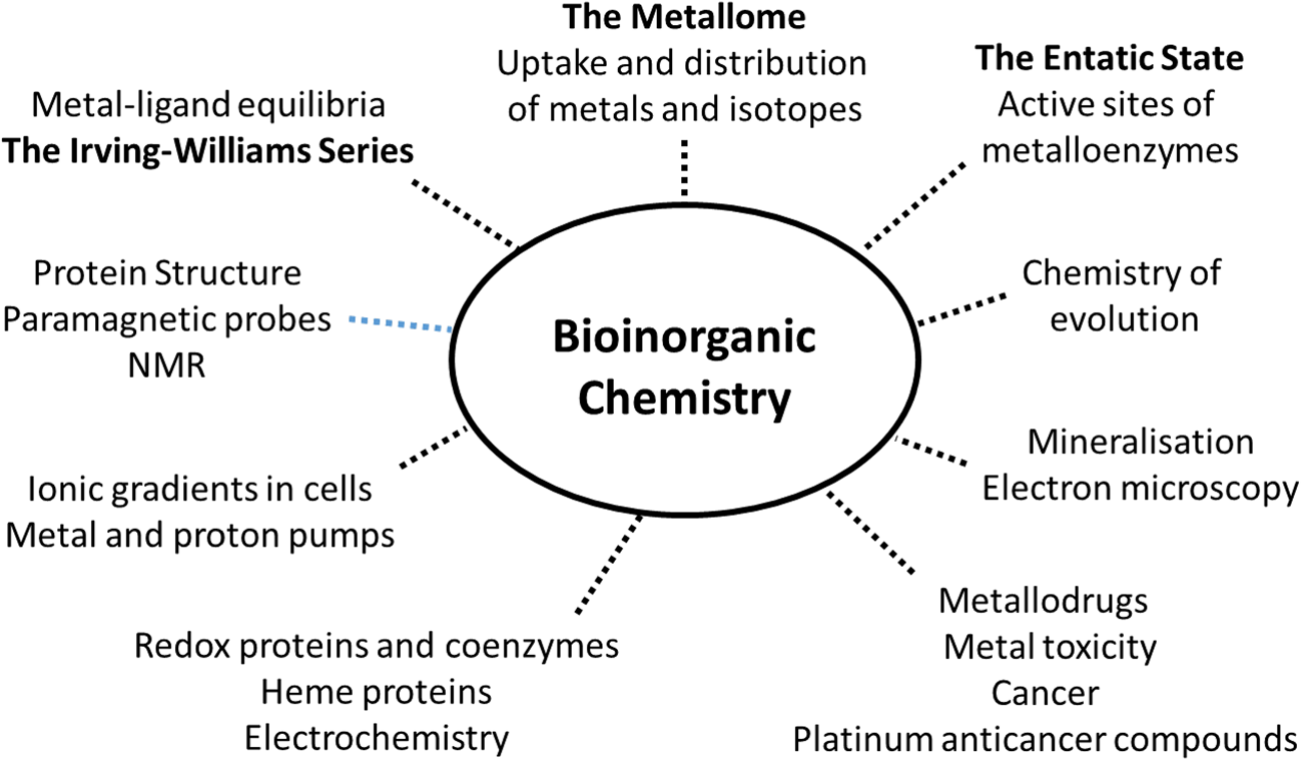
Some areas of research in which R. J. P. Williams made significant contributions, both experimental and theoretical

1. Metal enzymes and other metal compounds of biological interest are divided into two groups. One group contains a specific activating metal ion, whereas the other class of compounds, which is composed of enzymes only, requires a metal ion as a catalyst, several metal ions being somewhat differently active.
2. The formation of complexes between metal ions and simple ligands is a useful model for the formation of compounds between substrates, prosthetic groups, and the same metal ions.
3. Reasons are given for thinking that the stability of metal complexes usually falls into a fixed order of the metal ions such that.
  Mn^II^ < Fe^II^ < Co^II^ < Ni^II^ < Cu^II^ > Zn^II^
  and
  Ba^II^ < Sr^II^ < Ca^II^ < Mg^II^
  These orders are largely independent of the ligand.
4. However, certain metal ions can interact in a specific manner with special ligands. In particular, ferrous iron reacts strongly with aromatic nitrogen bases.
5. In a system in which there is an excess of ligands over metal ions and in which many different types of ligand are present, the different metal ions will not be found in the same kinds of complexes. It is suggested that a body fluid is such a system.
6. The formation of the different complexes of the metal ions is further restricted by the specific reactions mentioned in 4 above.
7. In a biological fluid the pH is fixed, and this pH will also limit the types of complexes that the various metal ions can form. The hydrogen ion is to be considered a competing metal ion and the hydroxyl ion a competing ligand.
8. The catalytic activity of metal ions in acid–base hydrolyses is interpreted in terms of intermediate complex formation. The mechanism of some of these reactions is discussed, as the reactions are very similar to certain enzyme reactions.
9. The interaction of metal ions with proteins is not very different from their interaction with simpler substances, but the large number of different coordinating groups available in a protein makes the interpretation of results difficult.
10. In proteins, some metals only occur in combination with certain co-ordinating groups; iron only occurs in the porphyrin nucleus.
11. The oxidation–reduction potentials of the haem proteins are discussed in the light of our knowledge of the potentials in model systems. The ferric haems are compared with chlorophyll.
12. The copper enzymes, such as haemocyanin, would appear to involve combination of the metal sulphur groups.
13. The specific interaction between phenolic or enolic groups and cupric ions is examined and an explanation of their importance is suggested in terms of the model systems.
14. The specific requirements for zinc and cobaltic ions are not understood, and only tentative suggestions can be made as to the nature of their compounds.
15. The less specific metal ion requirements of peptidases, phosphatases and carboxylases are discussed in terms of the models. The existing theories describing the function of the metal ions are criticized, and certain suggestions are made as to the importance of the different types of complexes formed by the different metal ions.

Items 10–12 in this list are wrong, but this is not unexpected given how little was known at the time about, e.g., redox-active proteins and enzymes containing iron-sulfur centres, but it is a surprisingly perceptive article, especially since it was created so soon after Bob became a regular inorganic chemist. He was given advice by many people to avoid an interest in matters related to biology; fortunately, he ignored this.

## Vitamin B_12_

After she had determined the crystal structure of penicillin [3], Dorothy Hodgkin tackled [4] that of Vitamin B_12_, and concomitantly Bob set about acquiring the physico-chemical data. It was relatively easy, owing to the intense absorption of the corrin, to study by electronic absorption spectroscopy, but other properties were more difficult to study. Nevertheless, the oxidation states of aquocobalamin and its reduced forms were determined [5] electrochemically as +3, +2, and +1. Though large amounts of relatively standard physical studies were published, the authors were particularly intrigued by the novel forms containing a Co–C bond, especially the coenzyme B_12_ and methylcobalamin. Thus these forms could be described as alkylated derivatives. But how were the coenzymes involved in the enzymatic reactions? Of course, it was obvious that when either coenzyme bound to an apoenyzme giving the various holoenzymes and in the presence of the appropriate substrate, the Co–C bond would transiently break–but the question was how?

One of the physical methods that had been used was electron paramagnetic resonance (EPR) spectroscopy [6], valuable for the low-spin d^7^ cobalt(II) derivatives. It was quite a surprise to find, in the holoenzyme diol-dehydratase, the system gave rise [7] to a striking EPR spectrum in the presence of the substrate *that was not obviously related to that of an unaltered Co(II) form*. Instead, it seemed more sensible to derive it from a Co(II) derivative of the corrinoid *and* the deoxyadenosyl fragment. Since that very early result, similar conclusions have been reached about many other B_12_-dependent enzymes.

The ability to investigate such systems has advanced nearly beyond measure with developments in genetic engineering, the sensitivity of EPR spectrometers, and, strangely enough, the advances in theoretical methods. For example, Jensen and Ryde performed [7] an interesting treatment of glutamate mutase and found that the bond dissociation energy of the Co–C bond is reduced by 135 kJ/mol in the enzyme, the effect owing to four terms: the restriction of the adenosyl radical to 4.2 Å of the Co ion, and the dissociated state is stabilised by 42 kJ/mol by electrostatic and van der Waals interactions. The other two terms come from the stabilization of the protein itself and of the remainder of the coenzyme. This information would have been immensely valuable to those considering the usefulness of another topic introduced by Bob, with his long-time colleague, Bert Vallee: entasis.

## The entatic state

It was dithizone that brought Bob and Bert together in the 1950s. Bob had used it as a reagent for metal ions in his investigation that ultimately resulted in the Irving-Williams series; Bert had found [8] it invaluable in his work on zinc-containing enzymes, which at the time were represented by two examples, carbonic anhydrase and carboxypeptidase. When they eventually met, they found they had many interests in common, but they approached any situation from quite different stances. Bob was a man driven by ideas, Bert was concerned with action, though his training as an analyst was of great value in the investigation of these metalloenzymes. Over the years, more and more such enzymes were isolated, but the subject moved faster in the case of haem enzymes since they were, along with haem proteins, greatly aided by their ready availability and, in many cases, by their intense colours. Fortunately, in the case of the zinc(II) enzymes, replacement by cobalt(II) gave rise to a Co(II) enzyme that was usually quite active. Bob had loved making lists of the properties of metal complexes since the 1950s and so, when he was on sabbatical leave with Bert, they presumably together made comparisons of the known metalloproteins and metalloenzymes with the properties of “model” complexes. The differences were great, so they proposed that in the biological complexes, the activity was associated with distortions, and they termed [9] the effect as being due to “entasis”; thus the protein or enzyme was in an entatic state. When more data become available, Bob returned [10] to the topic and gave a more convincing display of how proteins and enzymes exhibited conditions best described as entasis.

There has been some confusion over the meanings of *entasis* and a *rack* [11], and they are often treated as synonymous. That is not true. *Entasis* refers to a static locally energised condition of a group, a state; whilst a *rack* refers to a particular stretching machinery for a mechanically induced change of state of both protein and group interactions. An interesting paper [12] on “Recent advances in understanding blue copper proteins” attempts an explanation of “unusual” spectroscopic features such as the intense absorption band around 600 nm or an EPR spectrum showing a “weak” interaction between the unpaired electron and the nuclear moment of the copper(II). These were said to be associated with the presence of entasis or were rack-induced, *both* because of the imposition of an unusual geometric and electronic structure on the metal ion, thereby activating it.

## A change of scenery

Bob and HAOH belonged to the so-called Oxford Enzyme Group (OEG), which gave local scientists freedom to do interdisciplinary research. Most of the interest centred on X-ray diffraction, since that had recently been achieved with the structure of lysozyme [13], and on the NMR of proteins, since it was felt that this would eventually add some information on the movement within proteins. Bob decided to investigate a range of cytochromes [14]; I studied copper proteins [15]. At one of the informative meetings of the OEG, I asked a visiting lecturer, who had talked about electron transfer, about the electrochemistry of redox proteins. On hearing that this had not been reliably achieved, I decided to try. Eventually, with a young student, I succeeded [16], and so the field of protein electrochemistry was born.

The electrochemistry of enzymes was more difficult to achieve. Previously, seeking to make some use of the combination of electrochemistry and enzymes [17], Hill and colleagues had eventually found that the combination of a ferrocene and glucose oxidase provided an excellent electrochemical array to determine glucose. That it worked in whole blood [18], allowed the determination of glucose on a simple strip [19]. By 2013, over 25 billion such strips had been used. It is difficult to select the factors responsible for such success: the initial observation, the realisation that it could be the basis of a useful product, the search for proper, particularly financial, support, and the determination of those who sought to bring the product to the market place.

## Supramolecular chemistry

Bob’s interest in understanding the recognition of substrates by haem proteins led us to consider in the later 1960s the effects of weak non-covalent forces between molecules, in so-called charge-transfer and Mulliken complexes. This was “supramolecular chemistry” long before that phrase was coined. It led to the use of paramagnetic centres as probes for the determination of solution structures by NMR spectroscopy, initially using low-spin Co(II) in porphyrins [20, 21], and soon followed by the more general use of paramagnetic lanthanide ions for protein structure determination.

Bob’s studies on the relaxation properties of a variety of chelated gadolinium complexes [e.g., 22] inspired me (PJS) to investigate the potential uses of Gd(DTPA) derivatives of polysaccharides as contrast agents for magnetic resonance imaging (MRI) [23]. These were quite effective and attracted commercial interest from Amersham International and subsequently Guerbet.

## Platinum anticancer drugs

Bob played an important role in initiating the development of platinum anticancer drugs. The anticancer activity of the cisplatin family of drugs was discovered in the laboratory of Barnett Rosenberg at Michigan State University in the late 1960s [24]. Rosenberg had discovered some interesting effects of electrolysis products from Pt electrodes on the growth of bacteria [25], but Barney was a physicist and needed help to understand their mechanism of action and to elaborate on structure–activity relationships. In Bob’s own words, “Barney (Rosenberg) rang me up and said, ‘Bob, I am in difficulty, I don’t understand much about platinum chemistry. Will you be my consultant?’” [26].

The idea of using an inorganic compound, and especially a heavy metal complex, in therapy was not well-received by many people at that time, but Bob was always keen to promote and explore new ideas:“The first open [international] platinum meeting was run in Oxford in 1973, and basically I organized it at Wadham College and in the inorganic chemistry laboratory, which is totally appropriate, as cisplatin is inorganic” [26].

Bob corresponded with Rosenberg about the coordination chemistry of platinum. This played an important role in establishing a molecular basis for the effects Rosenberg observed. He had met Rosenberg earlier in the 1960s at a conference in California dealing with bioelectrochemistry and electron transfer, topics in which they shared a common interest. Bob’s DPhil student Andrew Thomson subsequently spent 2 years (September 1965 to August 1967) in Rosenberg’s lab synthesising, characterizing and testing platinum complexes for antibacterial and anticancer activity [27], and initiated work on cisplatin itself [26].

In particular they noted the high strength and relative inertness of the Pt-NH_3_ bonds compared to the Pt–Cl bonds, which could readily undergo aquation. Such an activation mechanism is thought to be the key step in the binding of cisplatin to DNA in cells. The chloride concentration outside cells is high (ca. 104 mM) and decreases in the cytoplasm (ca. 25 mM) and is even lower in the nucleus (ca. 4 mM) where activation by hydrolysis is favoured. There is good evidence that platination of adjacent guanines on DNA by the robust *cis*-{Pt(NH_3_)_2_}^2+^ unit to produce an intrastrand GG crosslink distorts the DNA, leading to HMG protein binding and the initiation of cell death by apoptosis (programmed cell death) [28].

Cisplatin has been joined in the clinic by carboplatin, a much less reactive complex with a chelated dicarboxylate ligand (CBDCA) replacing the two chloride ligands, and by oxaliplatin, in which the amine N ligands are now part of a chelate ring (1,2-diaminocyclohexane, DACH) and oxalate is also chelated. Release of CBDCA from carboplatin in cancer cells can lead to similar DNA lesions as cisplatin, whereas in the case of oxaliplatin, protein recognition of {Pt(DACH)}^2+^ is different from *cis*-{Pt(NH_3_)_2_}^2+^ and so lowers cross-resistance.

This early work showed that the *trans* isomer transplatin is inactive as an anticancer agent, and *trans* diamine complexes were not further explored until relatively recently when some *trans* complexes have been found to have good activity [29]. Interestingly in our (PJS) lab we find that photoactivated *trans* diamine Pt(IV) prodrugs are more active than the *cis* analogues [30].

The NH_3_ ligands in cisplatin can be labilised when the Pt-NH_3_ bonds are *trans* to ligands with *high* trans effects, such as the sulfur ligands in the amino acids methionine (thioether) and cysteine (thiol) [31]. Consequently the molecular pharmacology of cisplatin is more complicated than originally believed. Interactions of cisplatin with the copper influx transporter Ctr1, copper chaperone Atox1, and copper pumps ATP7A and ATP7B appear to be involved with Pt transport in cells and involve binding to methionine and cysteine residues. Further studies of such interactions may lead to a better understanding of the side-effects of Pt drugs and of resistance [32]. [Pt(l-methionine)_2_] is a metabolite of cisplatin [33].

## Organometallic anticancer complexes

Bob’s early interest in platinum drugs led me (PJS) to explore the broader area of transition metal anticancer complexes and their mechanisms of action. Initially we focussed on DNA as the target, since this is effective for cisplatin, but we attempted to create DNA lesions that were structurally distinct for those of platinum drugs, in the hope of circumventing cross-resistance (currently a clinical problem).

Our organometallic half-sandwich “piano-stool” Ru(II) complexes formed monofunctional as distinct from bifunctional (chelated) lesions on DNA formed by cisplatin and were not cross-resistant [34]. Our approach has kept in mind the principles that were prominent in Bob’s thinking: the need to consider not only the thermodynamics (equilibria) that might be involved in reactions with biomolecules (targets), but also the kinetics (rates of ligand exchange) as well as both metal and ligand-based redox reactions. He also stressed that reactions in cells seldom reach equilibrium. Although it is often useful to study “model” reactions in order to gain insight into biological mechanisms of action, we need to remember that cells are systems, rather than a collection of unconnected biomolecules. The biological system often functions under conditions far from equilibrium. Pumps, transporters, feedback loops, and other pathways often determine the flux of available ligands and electrons. To understand the mechanism of action of metallodrugs, many of which are multi-targeted, a “systems-pharmacology” approach is needed [35].

Half-sandwich organometallic complexes provide a rich platform for drug design. Their reactivity can be finely controlled by choice of the coordinated ligands. Introduction of an azopyridine ligand as an N,N-chelating ligand instead of ethylenediamine, for example, can render complexes inert and introduce redox mechanisms of action in cancer cells [36]. Some transfer hydrogenation catalysts appear to be able to carry out NAD^+^/NADH interconversions in cells [37, 38].

## The elements of life: natural selection of the elements

Bob was interested in the evolution of life’s chemistry. He analysed in detail the chemical speciation of the elements, the dynamics of element movement, and dependence on the state of the environment [39, 40]. In particular he incorporated the fundamental principles of inorganic chemistry into his reasoning, for example mineralisation and the familiar reactions associated with qualitative analysis (precipitation of metal sulphides etc.) [41].

Bob coined the term “metallome” so as to emphasise the critical role played by the distribution of metal ions in cellular compartments [42]:

“*The variety of paths which individual elements follow in any organism adds to the specific character of the organism. Clearly the paths have evolved to create an element distribution which we shall call the metallome”* [42].

*“The metallome must be recognised as a fundamental feature of a cellular compartment which is linked but not quantitatively to the proteome and the genome since it is related also to environmental availabilities and to energy supply”* [42].

*“The chemistry of evolution, according to me, is totally dependent on metal ions. You may not want to believe that, you may think it is totally dependent on DNA, but I believe that’s a thundering mistake”* [26].

*“There is no doubt therefore that the environment, non*-*metals and metal ions, interact with all the internal activity of cells, using external energy sources. The whole organism can be described as a sum of gene units of inheritance, not just DNA”* [43]

*“Do not forget that the whole environment and organisms are one system”* [44]

It is interesting to ask whether the elements we currently think are essential to man all have recognisable DNA codes, *i.e.* can be linked specifically to a protein that handles the element. A summary of current knowledge is presented in Table 1 [45]. There is a lot of uncertainty. Research on the essentiality of elements is not being pursued so vigorously today as it was 40 years ago. About 20 elements appear to be essential and most can be linked to DNA-coded proteins, but knowledge of how fluorine and vanadium are taken up and transported appears to be poor. About another six elements are potentially essential with, as yet, poor or little evidence of DNA codes.

**Table 1:** Essential elements for man *(adapted from ref 45)*

Atomic number	Element	Examples of genetic coding
1	H	Transmembrane proton pumps
6	C	Uptake of organic molecules (amino acids, fatty acids, vitamins etc.), regulation of CO_2_, carbonate, CO
7	N	Pathways for e.g. amino acids, nucleotides, NO synthases; conversion of NH_3_ into urea
8	O	O_2_ uptake, transport (haemoglobin) and storage (myoglobin), O_2_-sensor proteins, reduction of O_2_ to H_2_O (mitochondria), enzymes for O_2_^−^ and H_2_O_2_
9	F	F(^−^)/H(^+^) cotransporter or a F(^−^)/OH(^−^) antiporter
11	Na	Membrane pumps Na^+^/K^+^ ATPase
12	Mg	MAGT1, magnesium transporter 1
15	P	Kinases, phosphatases, nucleotides
16	S	Cys thiol/disulfide and Met in proteins; S^2−^ in ferredoxins
17	Cl	Cl^−^ channel: transmembrane conductance regulator (CTFR)
19	K	Membrane pumps Na^+^/K^+^ ATPase
20	Ca	Ca^2+^-sensor protein troponin; Ca^2+^-ATPase membrane pump; calmodulin transduces Ca^2+^ signals in cells
25	Mn	Most abundant Mn protein glutamine synthetase; Mn superoxide dismutase in mitochondria
26	Fe	Heme and non-heme Fe proteins
27	Co	Uptake and carrier proteins for vitamin B12
29	Cu	Cu^+^/Cu^2+^ proteins ca. 1 % of human proteome
30	Zn	Zn^2+^ proteins ca. 10 % of human proteome
34	Se	25 selenoproteins with redox and signalling functions
42	Mo	4 Mo enzymes (xanthine oxidoreductase and sulphite oxidase families); Mo cofactor
53	I	Thyroid hormones
Potentially essential elements		
14	Si	Role in bone mineralisation?
23	V	Role in phosphate biochemistry?
24	Cr	Influence on glucose metabolism?
28	Ni	Essential for some microorganisms Allergenic: MHCII-Ni-peptide recognition by T cells
35	Br	Essential for assembly of collagen IV scaffolds, and killing invading microorganisms as HOBr?
50	Sn	Essential for animal growth?

As an illustration, we discuss briefly the essentiality of the halogens. I (PJS) remember Bob emphasising in one of his lectures that several oxidation states of chlorine and iodine are important for their biological activity, not just the simple Cl(−1) and I(−1) ions. His overview of the solution-state (thermodynamic, equilibrium) chemistry of the halogens and other elements as portrayed by Oxidation State Diagrams (now often called Frost Diagrams) was particularly insightful, used to great effect in his textbook on Inorganic Chemistry in two volumes [46, 47], written jointly with Courtney Phillips and widely adopted by Oxford undergraduates. In contrast to the element-by-element or group-by-group approach of several inorganic other texts, it sought to provide an overarching view of the subject in a way that could reveal patterns and trends in a readily assimilable manner.

## The halogens as essential elements

There is no redox chemistry of fluorine in the body, just fluoride. Despite the widespread fluoridation of tap water with fluoride, which is known to be involved in calcium phosphate mineral formation, especially the enamel of teeth as fluoroapatite, relatively little is known about the biochemistry of fluorine. How is F^−^ transported across membranes? Since the pK_a_ of HF is low (ca. 3), it can exist in the stomach (where the pH drops to 1.5–3.5) as HF, and there appear to be specific HF transport proteins. There also appear to be F^−^/H^+^ cotransporter and F^−^/OH^−^ antiporter proteins.

Oxidised forms of Cl and I are, in general, strong oxidants, and while they are handled carefully in cells, often in membrane-bound compartments so as to avoid damage to natural cellular molecules, they are often used to damage toxins (e.g., invading organisms) deliberately within the compartment. For example, the abundant white blood cells neutrophils ingest pathogenic microorganisms and attack them with hypochlorite (bleach) formed from H_2_O_2_ and Cl^−^ by the haem enzyme myeloperoxidase (MPO). Activation of phagocytic leukocytes plays a key role in the immune response to invading pathogens. MPO can also generate HOBr, HOI, and HOSCN, which are also involved in the anti-bactericidal activity of neutrophils [48].

Bob was interested in the transport of the elements across cell membranes, the specificity of the channels and pumps, and the creation of gradients. Chloride concentration in body fluids is high (ca. 100 mM in blood) and decreases in the cytoplasm (ca. 25 mM) and cell nucleus (ca. 4 mM). Epithelial transport of Cl^−^ is mediated by the cystic fibrosis transmembrane conductance regulator (CTFR) a 1480 amino-acid cAMP-activated ATP-gated anion-channel glycoprotein. Mutations in the gene encoding CFTR can cause cystic fibrosis and a type of male sterility as a result of congenital absence of the vas deferens [49].

There are significant amounts of bromine in the body (ca. 0.2 g), e.g. in blood 3.2–5.6 µg/mL. Until a year ago (2014) we would have concluded that bromine is not an essential element for man and just noted that sodium bromide is an hypnotic, anticonvulsant, and sedative (Sedoneural) in medicine. Now it has been suggested that bromine is an essential trace element for all animals [50]. Bromide, on conversion to HOBr, is required for peroxidasin-catalyzed formation of sulfilimine crosslinks in a post-translational modification essential for tissue development within the collagen IV scaffold of basement membranes. Eosinophils (white blood cells) are components of the immune system and use eosinophil peroxidase, hydrogen peroxide H_2_O_2_, and Br^−^ to generate hypobromous acid (HOBr), a potent oxidant and part of the host defence against invading parasites and eosinophil-mediated tissue damage.

Iodide transport into follicular cells of the thyroid gland, the first step in the synthesis of thyroid hormones, involves a sodium/iodide symporter (NIS; sodium/iodide cotransporter) that transports two Na^+^ for each I^−^ into the cell [51]. However such transport is not well studied for other cell types. Oxidation of iodide by the enzyme thyroid peroxidase mediated by H_2_O_2_ is an important step for incorporation of iodine into thyroglobulin and the production of thyroid hormones tetraiodo prohormone thyroxine (T4) and triiodothyronine (T3).

## Metal isotope selection

Recently, in collaboration with Oxford geologists and others, Bob published some insightful papers on metal isotope fractionation in living systems.

*“Mass fractionation of transition metal isotopes can be induced to a considerable level by biological processes”* [51]

They concluded that organisms incorporate lighter isotopes of transition metals (e.g., of Fe and Cu) preferentially, and fractionation is controlled by redox processes. The implication of these results for the isotopic elemental composition of living things on geological timescales is thought-provoking.

Bob was inspiring because he was full of ideas and never afraid to enter areas of research where he or anyone else had not been before, especially using cutting-edge technology with potential for providing insight into problems posed by the inorganic chemistry of biological systems. Interdisciplinarity was key to his thinking, continually crossing the borders of chemistry, physics, biology, and medicine. The basis that he has established will make bioinorganic chemistry and inorganic biochemistry exciting areas of research for many generations to come.
